# Laser-Induced µ-Rooms for Osteocytes on Implant Surface: An In Vivo Study

**DOI:** 10.3390/nano12234229

**Published:** 2022-11-28

**Authors:** Vadim Veiko, Yuliya Karlagina, Ekaterina Zernitckaia, Elena Egorova, Maxim Radaev, Andrey Yaremenko, Gennadiy Chernenko, Valery Romanov, Nadezhda Shchedrina, Elena Ivanova, Boris Chichkov, Galina Odintsova

**Affiliations:** 1 Institute of Laser Technologies, ITMO University, Saint-Petersburg 197101, Russia; 2 Department of Dental Surgery and Maxillofacial Surgery, Pavlov First Saint-Petersburg State Medical University, Saint-Petersburg 197022, Russia; 3 Lenmiriot Dental Implant Prosthetics Manufacture, Saint-Petersburg 193079, Russia; 4 STEM, School of Science, RMIT University, Melbourne 3000, Australia; 5 Institute of Quantum Optics, Leibniz University of Hanover, 30167 Hannover, Germany

**Keywords:** biocompatibility, osseointegration, titanium implants, laser texturing, topography, in vivo, rabbit tibia

## Abstract

Laser processing of dental implant surfaces is becoming a more widespread replacement for classical techniques due to its undeniable advantages, including control of oxide formation and structure and surface relief at the microscale. Thus, using a laser, we created several biomimetic topographies of various shapes on the surface of titanium screw-shaped implants to research their success and survival rates. A distinctive feature of the topographies is the presence of “µ-rooms”, which are special spaces created by the depressions and elevations and are analogous to the µ-sized room in which the osteocyte will potentially live. We conducted the comparable in vivo study using dental implants with continuous (G-topography with µ-canals), discrete (S-topography with μ-cavities), and irregular (I-topography) laser-induced topographies. A histological analysis performed with the statistical method (with p-value less than 0.05) was conducted, which showed that G-topography had the highest BIC parameter and contained the highest number of mature osteocytes, indicating the best secondary stability and osseointegration.

## 1. Introduction

Dental implant survival largely depends on how delicately and correctly the artificial root is installed, as well as on home and professional control of the peri-implant microbiota, which is characterised by microbial diversity different from the that of the dental microbiota [1].

One of the most important roles in this process is the primary stability of the implants [2]. Over time, the primary stability of the implant decreases to almost zero due to bone resorption at the place of contact with the implant thread. Despite this fact, the implant remains in the jaw because new bone tissue is formed instead of destroyed bone tissue in the voids between the implant threads. The process of osseous fusion of bone tissue with the implant surface and restoration of the vascular system is determined by secondary stability [3,4]. Secondary stability occurs during the first minutes after implant placement and increases to the maximum at the time of complete osseointegration. It is essential for the long-term successful functioning of the installed implant and its long lifetime.

It is well-known that implants with a rough surface have significantly better secondary stability over implants with a smooth surface [5,6,7]. This happens because the contact area of cells with a rough surface is wider compared to that of a smooth one. In addition, not only surface roughness but also surface topography affects biocompatibility. There are different ways to modify topography of the implant, such as plasma spraying [8,9,10,11], sandblasting with large grit, [8,12,13] acid-etching (SLA), etc [14,15,16,17,18]. The above-mentioned have various disadvantages such as high probability of toxic effects on biological tissue [17], the residues of used acids remaining in the pores [19,20], low adhesion of plasma spray coatings to the surface, and low wear resistance [9,10,21].

One of the important scientific directions today is research aimed at finding new methods to improve integration properties of biomedical implants. Laser processing can be considered as one of the most promising methods. Laser processing of titanium dental implant surfaces is a one-shot manufacturing process that does not require consumables during production and allows control of the surface geometry at both micro- and nano- scales [22,23,24,25,26,27,28,29,30,31,32]. Aside from the capability of hierarchical relief creation by laser processing, this technology allows control of the array pitch, depth, and other parameters of the relief, including the surface roughness and material’s abrasion resistance, contact angle, biological properties (such as cell adhesion and biocompatibility, bacterial resistance), and rapid bone integration [33]. Formed nanoporous coatings on the titanium surface during laser ablation in air provide bioavailability at the early stages of osseointegration [23,25,28,29,32,34,35].

In recent studies on laser surface treatment of materials, it has been shown that the geometric shape of the surface, the size of pattern elements, and surface oxides affect adhesion [36,37,38], orientation angle [29,39,40], and differentiation and proliferation [37,41,42,43,44] of various types of cells. For example, it was shown during experiments on human osteosarcoma (HOS) cells [40] that a polished surface and a rough surface with non-periodic relief contribute to random orientation of cells, and it was found that cell spreading decreases with the increase of surface roughness. At the same time, on micro-grooved surfaces, the cells were oriented along the grooves, and a decrease in the distance between the grooves contributed to the enhanced orientation and attachment of cells. It was also concluded that smooth surfaces result in random osteosarcoma cell orientations and increased possibility of scar tissue formation [44]. In contrast, microgroove geometries have been shown to promote contact guidance of the cells, which leads to reduced scar tissue formation. In addition, discontinuous micro-grooved reliefs provide movement of the HOS cells. Within the micro-grid patterns, the cells seemed to be less mobile; they were found to attach to the tops of the bumps with relatively no alignment effects and to spread minimal distances from wherever they first landed. Cells’ guidance for human mesenchymal stem cells (hMSCs) and human osteoblasts has been confirmed in several works [29,36,39]. Groove dimensions (depth, width, and period) also affect cells’ guidance [45,46]. Animal experiments have demonstrated that laser-treated implants are able to promote better bone formation in comparison with machined implants [30,47]. Further, a laser-modified surface promotes higher biomechanical capacity, with interface characteristics more similar to those of the intact bone. Biomechanics were characterized by a failure pattern similar to bone fracture; in contrast, the machined implant was characterized by early plastic deformation [48]. It was found that when comparing laser-treated implants made of titanium alloy to zirconium implants, the influence of the surface topography was stronger compared to the effect of the surface elemental composition [49].

However, the question arises as to which particular surface topography is better for the osseointegration of the implants: discrete, continuous, or other? To answer this question, we utilised a biomimetic approach for designing various topographies.

**Figure 1:**
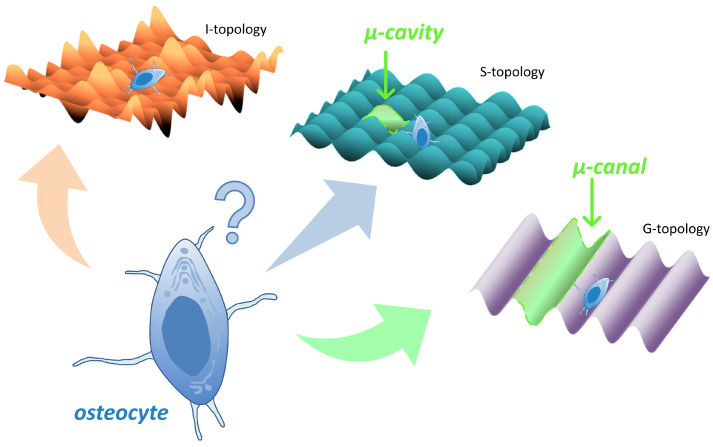
Design of topographies on the titanium implants’ surface of: irregular topography (I-topography) and two variants of topographies with μ-rooms (S-topography with μ-cavities and G-topography with μ-canals). This paper poses a question of what type of topography is the most preferable for osteocytes.

There are an infinite number of possible surface topographies, and it is obvious that it is not possible to conduct in vivo studies on all topographies in a row. We used the gradation of all possible topographies proposed in the work of C. Simitzi et al. [50], where all topographies are divided into two main groups: continuous (in the form of grooves) and discontinuous (in holes); we also supplemented them with a third type: topography with a random arrangement of elements. A biomimetic approach suggests having sizes of pattern elements commensurate with the size of the cells (we called these elements a μ-room). We designed two variants of μ-room topographies (Figure 1): S-topography, containing μ-cavities; and G-topography, containing μ-canals. We also included a third type of topography in the study: irregular topography (I-topography in Figure 1), which is the most-studied today. The results of in vitro study of these topographies are presented in the work of Veiko et al. [41].

In this article, we present the results of an in vivo study of implants with these topographies in order to answer the question of whether a μ-room is needed for the comfortable life of bone cells. We performed a comprehensive sufficient histological and histomorphometric analysis, which previously has not been demonstrated.

## 2. Materials and Methods

### 2.1. Implants

Screw-shaped implants were manufactured from Ti-6A1-4V cylindrical bars by Lenmiriot Dental Implant Prosthetics Manufacture (St. Petersburg, Russia). The diameter of screw-shaped implants is 3.5 mm, and the length is 6 mm. The initial surface of screw-shaped implants (before laser processing) was only machined during the process of micromilling; the corresponding roughness was Ra = 0.6 ± 0.02 µm (measured with profilometer Hommel Tester T8000 (Jenoptik, Jena, Germany)).

### 2.2. Formation of μ-Rooms for Osteocytes on the Titanium Implant Surface

**Figure 2:**
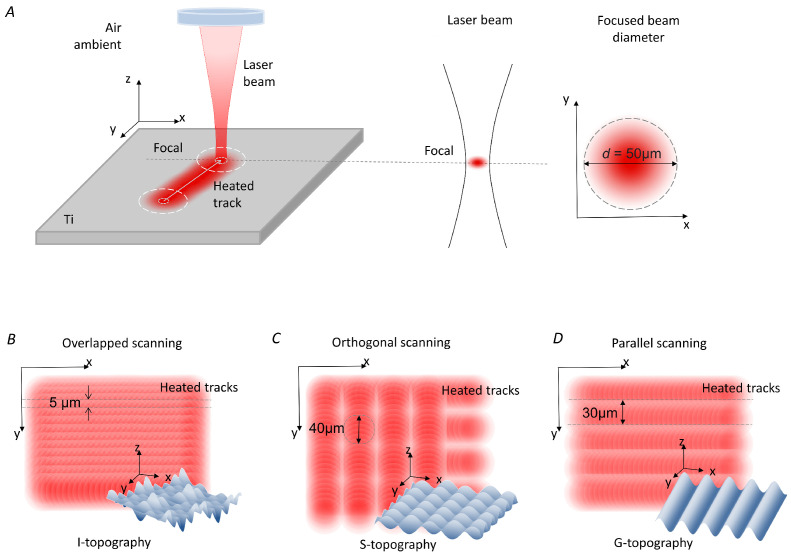
Schematic representation of the laser processing process. (**A**) Illustration of the laser titanium surface treatment at the optical system focus with a laser spot of 50 μm diameter with a Gaussian intensity distribution over the beam cross section; scheme of surface scanning by laser pulses and a model of the resulting topography of (**B**) “irregular structure” I-topography, (**C**) “slots” S-topography, and (**D**) µ-room-shaped “grooves” G-topography.

The surface of the screw-shaped implants was processed by a commercially available pulsed ytterbium fibre laser (Laser Center Co., Ltd., Russia) with the wavelength of 1.064 μm. Pulse duration τ used in the experiments was 100 ns; the spot diameter *d* in focus was 50 μm (Figure 2A). The laser complex was equipped with a rotator (ISEL ZD2030, Eichenzell, Germany) for processing the full surface of the screw-shaped implants.

Four groups of implants were created, differing in surface topography. The first group, named “Control”, consisted of implants with non-treated surfaces, i.e., the surface was only machined during the process of micromilling. The other three groups of Ti implants had their surfaces modified by laser structuring and were named “irregular structure” I, “grooves” G, and “slots” S. These topographies were formed in three different ways. The I-topography was created by sequential surface scans with laser tracks recorded with greater than 90% laser spot overlapping in the *x*- and *y*-axes (Figure 2B). Laser parameters for the I-topography were intensity *I* = 7 ·$10^{7}$ W/cm${}^{2}$, laser spot overlapping along the *x*-axis of *M${}_{x}$* = 93%, and laser spot overlapping along the *y*-axis of *M${}_{y}$* = 90%. A two-pass scan was used to form the S-topography (Figure 2C). After the first pass by laser pulses with intensity *I* = 7 ·$10^{7}$ W/cm${}^{2}$, overlapping *M${}_{x}$* = 99% and non-overlapping *M${}_{y}$* = 33% parallel grooves were formed on the entire surface. The second pass recorded grooves with the same *M${}_{x}$* and *M${}_{y}$* values but in the orthogonal direction. In areas where repeated impulses hit, depressions in the form of dimples were formed. The creation of the G-topography involved three stages (Figure 2D). The first pass was the formation of parallel grooves with a period of 90 μm (*M${}_{x}$* = 99% and *M${}_{y}$* = 0%). In the second pass, the same grooves were recorded but with an offset of 30 μm along the *y*-axis relative to the grooves formed after the first pass. During the third pass, the same grooves were recorded but with an offset of 30 μm along the *y*-axis relative to the grooves formed after the second pass. Thus, µ-rooms in the shape of periodic grooves 20 to 50 µm wide and deep were formed on the surface. Laser intensity for the G-topography was *I* = 5 ·$10^{7}$ W/cm${}^{2}$. A total of 15 laser-structured implants of each type were created. Each third implant of each topography type was technically controlled via optical microscopy using a ZEISS Axio Imager A1 microscope (Carl Zeiss, Oberkochen, Germany). The presence of I-, S-, and G-topographies on all parts of the implant was monitored. Slight deviations in the fin and groove sections were accepted.

Scanning electron microscopy (SEM) analysis was performed using a Zeiss Merlin microscope (Zeiss, Germany) equipped with an additional device for X-ray microanalysis: an Oxford Instruments INCAx-act.

### 2.3. Clinical Procedure

During all surgical procedures, the animals used in experiments were preliminarily prepared for premedication (atropine s/c 10–15 min before premedication at a dose of 0.1 mg/kg); then, the premedication was carried out: rometar (trade name: injection solution Xila, Interchemie, Netherlands) s/c, i/m at a dose of 4 mg/kg, ketamine s/c, i/m at a dose of 10–15 mg/kg, and droperidol 0.25% solution s/c, i/m at a dose of 2.5 mg/kg. Injection anaesthesia was performed with ketamine (5–10 mg/kg IV) and rometar (2–4 g/kg IV) until the lingual, swallowing, pedal, and corneal reflexes disappeared. A maintenance injection was given to animals every 10–15 min depending on the animals’ response to anaesthesia. An intravenous catheter was inserted in the cephalic vein, and propofol was infused at a slow constant rate of 0.4 mg/kg/min. Local infiltrative anaesthesia was administered at the surgical sites.

Fifteen female rabbits of one year of age, each weighing 4–5 kg, were used in the present study. In each tibia of the rabbit, two dental implants were installed, that is, four implants per animal. A total of 60 implants were installed. A longitudinal incision was made in both tibiae of the rabbit. The skin with subcutaneous fat was exfoliated, the muscles were exfoliated, and a bed for the implants was formed by successively changing the cutters using a physiological dispenser with physiologically soluble NaCl 0.9%. Implants were installed using an adapter and a torque wrench using insertion torque of 20–30 N/cm. The wounds were washed with gentamicin solution. Sutures were applied (suture material: vicryl 5.0; Ethicon, Summerville, NJ, USA).

In the first 24 h after surgery, a general check of the condition of the rabbits was carried out. After the operation, the animals were given heating pads. Animals were observed until all reflexes appeared. After the animal had taken its natural anatomical position in space (lying on its paws and holding its head), it was transferred to a permanent cage.

During the first week after surgery, the animals received antibiotics and analgesics: gentamicin (5–8 mg/kg IM, once a day) and Ibuprofen (600 mg, three times a day) via the systemic route. Animals were fed a soft diet for 14 days followed by a normal pellet diet. All animals survived the postoperative period without any complications.

### 2.4. Surgical Preparation

#### 2.4.1. Sacrifice

**Figure 3:**
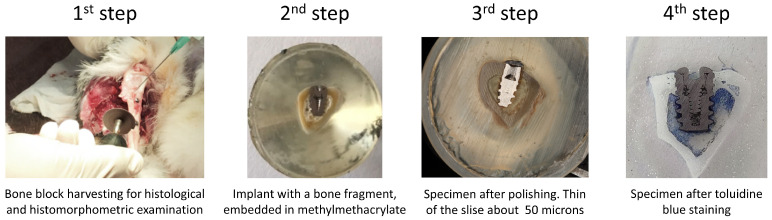
The histological preparation steps: 1—extraction of an implant with bone fragment; 2—cross-section of an implant with a bone fragment embedded in methylmethacrylate; 3—the sample after polishing; and 4—toluidine-blue-stained sample.

Seven animals were sacrificed after 1.5 months, and the remaining eight were sacrificed after a 3-month healing period. For euthanasia, the veterinary surgeon administered pentobarbital sodium (Abbott Laboratories, Chicago, IL, USA), subsequently perfused with a fixative (4% formaldehyde solution), through the carotid arteries. Rabbit tibias were en bloc dissected, and the surrounding soft tissues were detached (the first step in Figure 3). No implant failure was detected over the study period (1.5 or 3 months). The soft tissues were healthy and without signs of inflammation, hyperemia, swelling, or oozing.

#### 2.4.2. Histological Preparation and Examination

The histological and histomorphometric studies were carried out at the Center for Collective Use of Scientific Equipment “Cellular and Molecular Technologies for Studying Plants and Fungi” of the Botanical Institute V.L. Komarov RAS.

Regular histological methods of preparing a histological specimen, such as embedding in paraffin, celloidin, or polivax, do not allow obtaining a specimen containing both a titanium implant and the adjacent bone [51,52]. Therefore, we used special protocols for sample preparation consisting of impregnation and pouring of the test material into plastics and synthetic resins, as has been demonstrated in some studies [53]. This technique allows the obtaining of 100–200 µm primary sections, from which further histological sections with a thickness of 10 to 50 µm can be obtained.

The samples were fixed in formalin and dehydrated in a graded series of ethanol for 15 min each and dried with acetone of 30%, 50%, 70%, and 90% for 15 min each, then with 100% acetone for 30 min. The samples were then embedded in methylmethacrylate (Technovit 7100^®^, Heraeus Kulzer, Wehrheim, Germany). Using a micro-cut diamond bur (St. Petersburg State University, Institute of Earth Science), samples were cut in a vestibulo–lingual direction into 100 μm thick sections along the axis of each implant (the second step in Figure 3). These sections were ground down to 50–80 μm thickness using extra-fine paper discs with 2000-grain granulometry (the third step in Figure 3). A longitudinal section was made through the middle of the implant to form three sections. Toluidine blue staining was applied (the fourth step in Figure 3).

The sections were studied and analysed under light microscopy (Olympus BX 61, Hamburg, Germany and Zeiss Axio Imager A1, Carl Zeiss, Oberkochen, Germany). Histomorphometry was performed with a video camera (Sony 3CCD, Berlin, Germany) of 70× magnification. Images were digitalized (Axiophot-System, Carl Zeiss, Oberkochen, Germany), and benchmarks were established.

### 2.5. Statistical Analysis

The images of the obtained histological sections for G-topography, S-topography, I-topography, and control samples, were studied, based on which the parameters of osseointegration BIC and FIC, the number of cells, the surface area under cells, and average cell size were calculated. To assess the survival rate, samples were studied with different durations of the implant being in the bone: 1.5 months or 3 months of healing.

The parameters BIC (bone-to-implant contact) and FIC (fibrous-to-implant contact) were calculated as the ratios of the lengths for the areas at the site of contact of the implant surface with the bone tissue and granulation tissue, respectively, to the entire border of the bone implant (blue and yellow in total) at a distance of 800 µm for each sample. The calculations were carried out by the Digimizer Image Analysis Software.

To calculate the number of cells, the surface area under cells, and average cell size, three areas adjacent to the implant, 200 × 100 μm size and 2 ·$10^{4}$ μm${}^{2}$ area, were isolated on each sample. The boundaries of bone cells were manually highlighted in Adobe Photoshop to exclude contrast areas that were Haversian canals or titanium sawdust and to not miss parts of cells that were defocused and thus not identified by the software. The cell area and the number of cells were defined in the ImageJ program using the “Analyze Particles” function. Then, the area of cells was calculated as the ratio of the cell area to the number of cells.

A statistical test was carried out with Student’s parametric test for independent series, where the control group was untreated samples and the test group was laser-structured samples. The test was carried out solely between samples of the same time interval (1.5 months or 3 months). For the correct use of the criterion, the data were checked for normality of the mean values within all samples. The test was conducted with two levels of statistical significance at *a* = 0.05 and *a* = 0.01, which considers the correction for multiple-hypothesis testing. Based on the results of calculation of osseointegration parameters, *p*-value < 0.05 (*) and *p*-value < 0.01 (**) were obtained.

Data visualization and statistical analysis were performed using GraphPad Prism software 8.01 (GraphPad Software Inc., La Jolla, CA, USA).

## 3. Results

The experimental stages were the following: creation of four groups of implants (control, “irregular structure” I-topography, “slots” S-topography, and μ-rooms-shaped “grooves” G-topography), surgical procedures of implant integration into rabbits’ tibias, extraction of implants with the surrounding bone tissues, and subsequent histological analysis.

### 3.1. Laser Structuring of Implants

**Figure 4:**
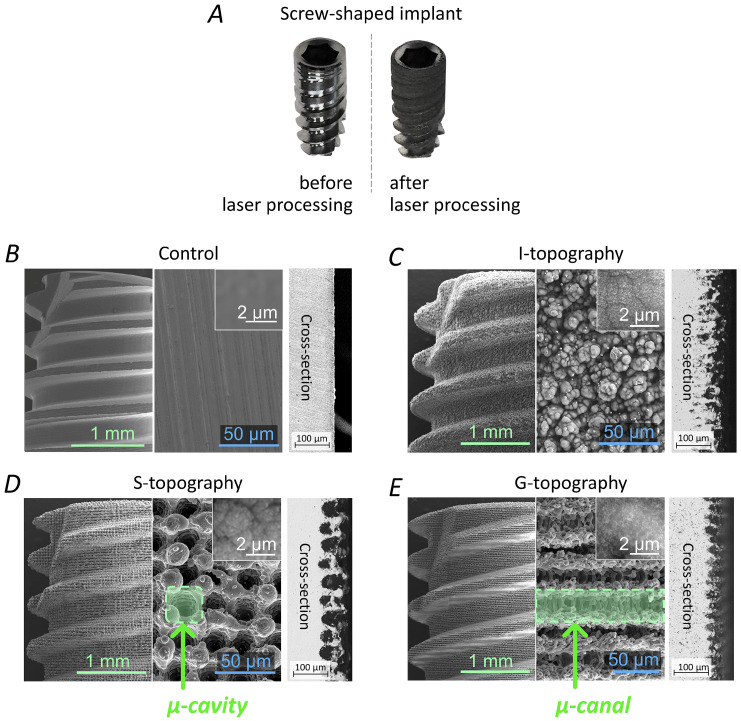
Images of implants. (**A**) Implant before and after laser texturing; SEM-images of the laser-textured implants: the profile, magnified view, and cross-section of (**B**) untextured control group, (**C**) “irregular structure” I-topography and µ-rooms-shaped (**D**) “slots” S-topography and (**E**) “grooves” G-topography.

Three types of topographies (“irregular structure” I-topography, “slots” S-topography, and μ-rooms-shaped “grooves” G-topography) were created using laser structuring. Optical images of unstructured and laser-processed implants are shown in Figure 4A. SEM images are shown in Figure 4B–E. The obtained surface topography has a special feature—in addition to first-order microstructure formation, the size of which was ≥10 μm, second-order microstructures (<10 μm) were formed on laser-structured sample surfaces. Therefore, the surface area was significantly increased, and, consequently, the potential area of cell contact with the surface was also increased. The height of laser-induced microstructures did not exceed 100 µm (see cross-sections in Figure 4C–E). The average size of I-topography elements was 15.5 ± 10.8 µm. The S-topography period was 49.5 ± 4.6 µm. The G-topography period was 30.2 ± 2.4 µm. The surface of the first and the second order structures were covered with a nonporous layer with a pore size of less than 150 nm. A more-detailed description of this layer is given in our previous work [41]. The presence of nanoporous topography promotes protein adhesion, which plays an important role in the early stages of implant survival.

The elemental composition of the near-surface layers of the samples was investigated by EDX analysis before and after laser structuring, with the typical error of EDX measurement being about 0.5%. As can be seen from the results presented in Table 1, laser treatment led to significant enrichment of the surface with oxygen compared to the nontreated samples. The oxide percentage for laser-treated samples varied within a ratio from 16.67 to 27.41 wt%. The variation in the percentage of oxygen in the near-surface layers is probably related to the phase transition during laser treatment. A more-detailed study of phase transformations in near-surface layers as a result of laser treatment under various exposure modes is a topic for a separate study. We assumed their chemical compositions to be analogous, as topography formatting was conducted under similar conditions.

### 3.2. Histological Analysis

**Figure 5:**
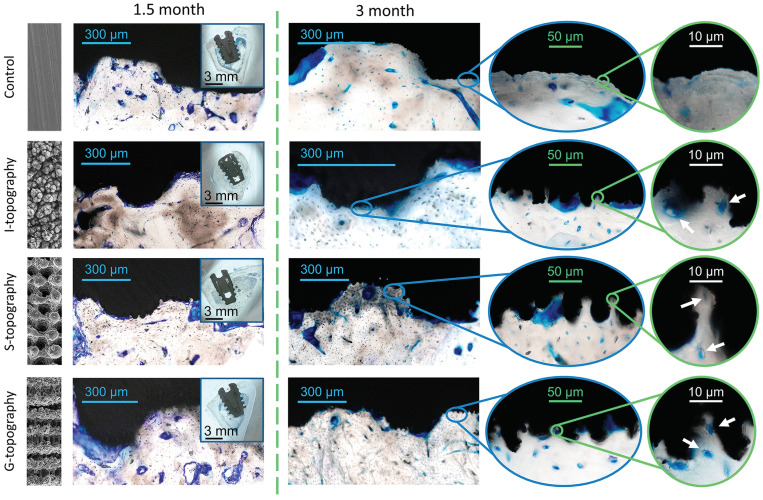
Optical images of histological sections of untextured (control) and laser-textured implants: 1.5-month-old (1st column) and 3-month-old (2nd column). White arrows point to the osteocytes in the μ-cavities and μ-canals.

Optical images of histological samples are presented in Figure 5. Histological samples of the polished implants (control group) that remained installed for a period of 1.5 months present a fragment of a compact bone with an uneven arrangement of osteocytes and Haversian canals of different sizes and shapes. Along the fragment edges, bone tissue can be found, as evidenced by the larger size of the osteocytes and the darker background of the matrix. Their surfaces are covered with basophilic tissue of various thickness. This tissue is most likely fibrous and contains detritus. For the samples from the control group that remained installed for 3 months, it seems that the bone fragment is already represented by a more mature, compact bone. Young bone tissue is formed on its surface with the ongoing maturation processes.

The 1.5-month-old samples of the I-topography are represented by compact bone with an uneven edge covered with cellular fibrous tissue of uneven thickness. Under the tissue, there is a layer of darker bone tissue with a large number of evenly spaced bone cells, namely osteocytes. In the thickness of the fragment, Haversian canals of different sizes can be seen with vessels that are located unevenly in the bone matrix. Osteoblasts are seen in places on the canal walls. Osteocytes are found throughout the entire bone fragment. They are formed by viable bone with signs of recent bone formation and phenomenon of compaction, which is maturation of young bone into mature lamellar bone. The 3-month-old samples show a mature lamellar bone with irregularly spaced Haversian canals of different diameters. This indicates the restructuring processes in the bone tissue. Osteocytes are also distributed uniformly over the studied fragments. Processes of bone cells connecting to each other are well contoured.

The 1.5-month-old samples of the S-topography are represented by mature compact bone with evenly spaced osteocytes and unevenly spaced Haversian canals of different sizes and shapes. Younger bone tissue is located at the edges of the studied areas because the size of osteocytes is larger there, and the background of the matrix is darker. Their surfaces are covered with basophilic tissue of varying thickness. It is difficult to determine the nature of the tissue—it may be fibrous mixed with detritus. Three months after implantation, mature compact bone is in contact with the surface of the S-topography implant, on the surface of which young bone tissue is still forming with ongoing maturation processes.

Compact lamellar bone was identified on 1.5-month-old samples with the G-topography. On 3-month-old samples, unevenly spaced Haversian canals of normal structure and osteocytes in the lamellar bone are observed. Some osteocytes do not contain nuclei. In general, the formed bone tissue, which adheres tightly to the surface of the implant, contains mature osteocytes. Single osteocytes are located in the grooves, as if in lacunae.

### 3.3. Osseointegration Parameters

**Figure 6:**
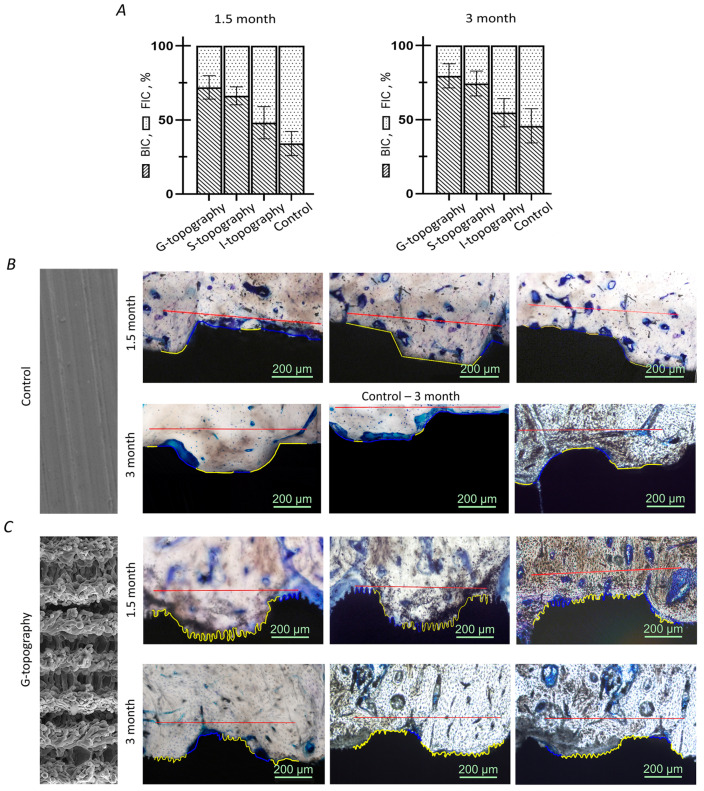
(**A**) Osseointegration parameters BIC (bone-to-implant contact) and FIC (fibrous-to-implant contact) for 1.5- and 3-month results (**B**) the control group and (**C**) the G-topography; yellow line is the length of the areas at the site of implant surface contact with the bone tissue, blue line is the implant surface contact with granulation tissue, and red line is a distance of 800 µm for each sample.

The results of calculating BIC and FIC parameters are presented in a diagram (Figure 6A). The parameters are given as the percentage of the total length of the bone–implant interface. The lines indicating areas at the site of contact of the implant surface with the bone tissue and granulation tissue to the entire border bone implant are presented in Figure 6B for the control group and Figure 6C for G-topography.

At 1.5 months after implant installation, the BIC parameter of dental implants with G-topography was 72%, with S-topography—66%, and with I-topography—48%. At 1.5 months after the surgery, the FIC parameter of dental implants with G-topography was 28%, with S-topography—34%, and with I-topography—52%. The BIC parameter of the control group of samples at this period was 34%, and the FIC parameter was 66%.

The 1.5-month results show that the newly formed bone tissue’s contact with the implant surface (BIC) is larger than the fibrous tissue’s contact with the implant surface (FIC) for the G-topography and for the S-topography. However, bone tissue contact (BIC) is smaller than the fibrous tissue contact (FIC) for the I-topography and for the control sample.

Three months after the implant installation, the BIC parameter of dental implants with G-topography was 80%, with S-topography—74%, and with I-topography—59%. At 3 months after the implant installation, the FIC parameter of dental implants with G-topography was 20%, with S-topography—26%, and with I-topography—41%. The BIC parameter of the control group of samples at this period was 46%, and the FIC parameter was 54%. For the 3-month results, this trend continues with BIC being larger than FIC for the G-topography, the S-topography, and also for the I-topography, but vice versa for the Control sample. A decrease in the FIC parameter with an increase in the duration of the implants’ stay in the bone shows gradual osseointegration of each type of implant. However, the G-topography shows the best results in terms of the BIC parameter, which indicates a high implant survival rate.

**Figure 7:**
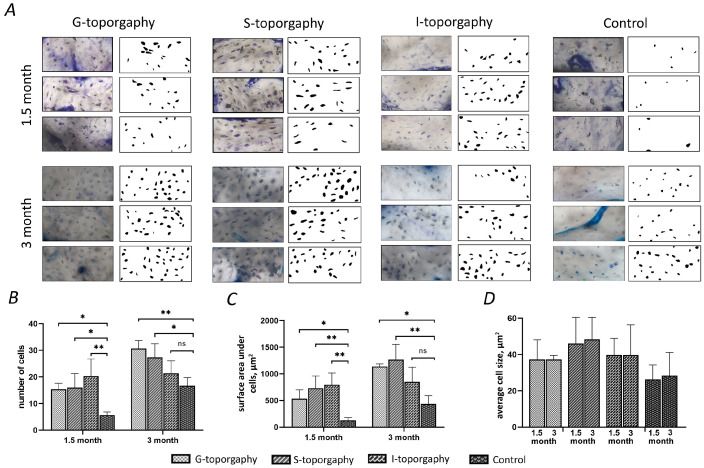
Osseointegration parameters for 1.5- and 3-month results: (**A**) images of bone tissue and the corresponding processed images of osteocyte; (**B**) the number of cells; (**C**) the area occupied by cells (cell area); and (**D**) average cell size; (*) indicates *p*-value < 0.05, (**) indicates *p*-value < 0.01.

The number and size of osteocytes were studied by processing the images of histological sections. Images of bone tissue and the corresponding processed images of osteocytes are shown in Figure 7A. For calculations, both newly formed areas of bone tissue (between the implant coils) and old bone tissue located around the hole for the implant) were used.

The obtained values of the number of cells in the selected areas of the bone are shown in the histogram (Figure 7B). At 1.5 months after implant installation, the number of cells in the implant area with G-topography was 15, with S-topography—16, with I-topography—20, and for the control group (without laser structuring)—5. At 3 months after implant installation, the number of osteocytes in the area of implant with G-topography increased to 31, with S-topography—27, with I-topography—22, and for the control group (without laser structuring)—19. The numerical values of the surface area under cells of 1.5-month-old bone tissue occupied by osteocytes were as follows (Figure 7C): G-topography—565 µm${}^{2}$, S-topography—731 µm${}^{2}$, I-topography—797 µm${}^{2}$, and control group (without laser structuring)—127 µm${}^{2}$. The numerical values of the surface area of 3-month-old bone tissue occupied by osteocytes were as follows: G-topography—1139 µm${}^{2}$, S-topography—1336 µm${}^{2}$, I-topography—877 µm${}^{2}$, and control group (without laser structuring)—527 µm${}^{2}$.

The average cell size in µm${}^{2}$ was calculated by dividing the surface area under cells by their number (Figure 7D). The average cell size on the sample with G-topography was 37 µm${}^{2}$ in both 1.5-month-old and 3-month-old bones. The average cell size on the S-topography specimen was 46 µm${}^{2}$ in 1.5-month-old bone and 49 µm${}^{2}$ in 3-month-old bone. The average cell size on the I-topography specimen was 39 µm${}^{2}$ in both 1.5- and 3-month-old bones. The average cell size on a sample from the control group was 25 µm${}^{2}$ in 1.5-month-old bone and 28 µm${}^{2}$ in 3-month-old bone.

An increase in the number of cells and the area under cells in 3-month-old samples suggests a decrease in resorption sites in the tissue due to the formed reconstructed bone tissue. A decrease in the resorption site number in the period from 1.5 to 3 months can be assessed by the histological section in Figure 5. All samples showed good results, which indicates that the implants are not cytotoxic.

### 3.4. Resonance Frequency Analysis

After sacrificing animals, the stability of the dental implants was assessed using resonance frequency analysis (RFA) on an Osstell ISQ device (“0” units correspond to the minimum stability, “100” units—to the maximum). RFA shows that the differences in the parameters of osseointegration are associated precisely with the difference in the surface topography of the implants.

**Figure 8:**
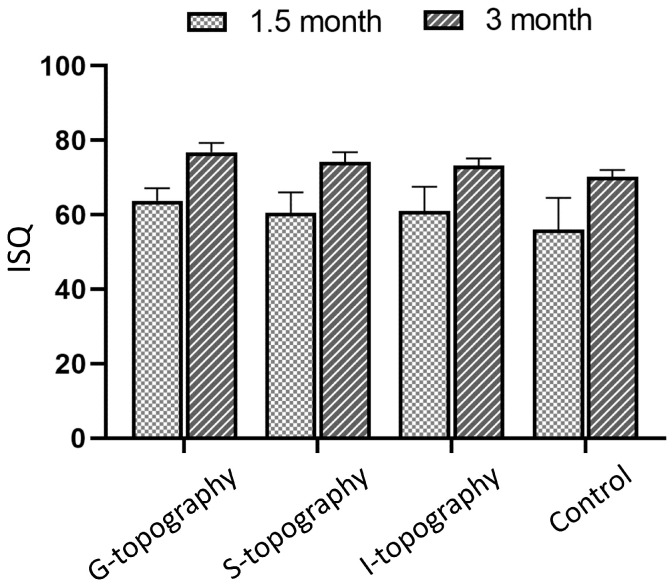
Results of assessing the stability of the implants using the Osstell ISQ device: distribution of the stability index of implants for 1.5 and 3 months.

For 1.5-month samples, the average value of the stability index of dental implants with a laser-structured surface (G-, S-, and I-topographies) was 60.5 units or above, which, according to the device manufacturer, is considered a good indicator of implant stabilization. At the same time, the value for untreated implants was 56 units, which is considered low stability. For 3-month samples, the average value of the stability index lies in the range of 73.2–79.8 units, which corresponds to high stability. The stability index of implants with an untreated surface is 70.2, which is lower than for samples with laser-induced topographies. The results are presented in normalized form in Figure 8.

## 4. Discussion

In previous research, animal experiments demonstrated that laser-treated implants were able to promote better bone formation in comparison with machined implants [30,47]. The difference in the result of these two types of treatment lies in the diverse chemical composition and in the diverse topography of the treated surface. Considering these two factors as potentially influencing osseointegration, it was found that the influence of the surface topography was stronger compared to the effect of the surface elemental composition [49]. The results of our work confirm the influence of surface topography on osseointegration in vivo. In terms of osseointegration potential, an ordered surface topography is better than a disordered one, and a continuous groove topography is better than a discontinuous alveolar topography. This is evidenced by the bone-to-implant Contact (BIC) value. Maximizing the BIC for osseointegration is a goal of treatment of implant surfaces [54]. In addition, the observed decrease in the FIC parameter with increased duration of the implant in the bone from 1.5 to 3 months shows increasing osseointegration.

It is essential to conduct a deeper analysis of the influence of surface topography on osseointegration potential. Thus, in this work, in addition to the standard parameters for determining the level of implant osseointegration (BIC and FIC), osseointegration was assessed by calculating the number of cells, the size of the area occupied by cells, and the average size of cells. These parameters were selected from the morphometric nomenclature for assessing the osseointegration of intraosseous implants [52], which is based on the recommendations of the American Society for Bone and Mineral Research. The calculation of several parameters allows getting a more generalized picture of the bone remodelling process.

We analysed the areas of not only the newly formed bone in the implant recesses (zones between the implant coils) but also the areas of the “old” bone tissue that formed the walls of the implant hole prior to its installation. According to the increase in the number of cells (Figure 7B) and the surface area under cells (Figure 7C) on histological sections of 3-month-old samples, there was a decrease in resorption areas in old bone tissue; therefore, an ongoing bone remodelling process can be assumed [55]. In this case, implants with G-topography and S-topography show the best results. Implants with different structures probably exert different stress on the bone, causing microdamage to tissue. This microdamage, in turn, causes the formation of resorption cavities and, consequently, bone remodelling.

Such a large difference in cell size for each topography (Figure 7D) can be explained by the ongoing remodulation of bone tissue. Bone during remodulation is more fragile and less durable [56], so it is worth considering the time that was spent on rebuilding the bone tissue from “old” to “new”. Taking this into account, the variation in osteocyte size for G-topography is significantly smaller compared to other topographies (Figure 7D). This indicates the completed restructuring processes in the tissue, and hence the strength of the bone and the stability of the implant. It is also worth noting that osteocyte cells are present in the G-topography grooves (Figure 5), which confirms that this surface is the most preferable for stable bone–implant contact.

The results obtained in this work correlate well with our results from in vitro study on mesenchymal stem cells [41]. In the in vitro study, we found that cell proliferation is better on micro-grooved topography. In addition, it was shown that surface topography affects the orientation of cells in space. In the case of grooves, cells grow along them.

We must consider that the current study has some limitations, such as the differences in the topographies of the parts of the threads of implants that appear to contribute to osseointegration. In particular, the topography on inclined threads may differ slightly from the topography on straight threads. The reason for this is that the height of the thread is greater than the caustic of the laser beam, and also because of the inclination of the surface of these parts of the thread, which are not perpendicular to the direction of incidence of the laser beam. Nevertheless, the calculation of osseointegration parameters was carried out in the areas of cross-sections of implants containing parts of implants, both straight and inclined. Consequently, the obtained numerical values of the osseointegration parameters are averaged. The next limitation is connected to the chosen rabbit animal model, which is commonly used in such research [57]. However, it should be noticed that the osseointegration results of the implant can be different for other animal models or for human implantation. Another limitation is associated with long-term implant survival. In our research, the implant osseointegration was studied for 1.5- and 3-month periods. A longer observation period (6 months and longer) is necessary in order to obtain results of long-term implant success.

Future clinical studies are necessary, such as the role of this implant topography in bacterial contamination and cases of peri-implantitis, how the results may be affected through scaling and root planing methods, and topical applications of antibiotics, ozone, photodynamic treatment, and probiotics [58].

## 5. Conclusions

This paper presents the results of in vivo study of the biointegration of dental implants with a laser-structured surface. Using the advantages of laser ablation, such as chemical (lack of reagents) and physical (lack of abrasive particles) process purity, non-contact exposure, one-stage surface processing with the formation of high roughness, and a biocompatible oxide layer, we created three different topographies on the surfaces of titanium screw-shaped implants. S-topography containing μ-cavities and G-topography containing μ-canals have a distinctive feature: the presence of “μ-rooms”; “μ-rooms” are special spaces created by the depressions and elevation and are comparable to the osteocyte size. The third type of topography is the I-topography, which has irregular structure.

Four groups of implants (three laser-structured with different topographies and one control group) were implanted into tibias of rabbits. The implants remained in rabbits for 1.5 or 3 months. After sacrifice, histological analysis was carried out with calculation of the BIC and FIC parameters, the area occupied by cells, the number of cells, and the average cell size.

The best osseointegration in the rabbit tibias after three months was demonstrated by an implant with a surface G-topography with a width and depth of about 20–50 µm (commensurate with the size of cells) in the form of periodic grooves. The study of histological samples of this topography showed that the formed bone tissue, which adheres tightly to the implant surface (BIC = 80%), contains mature osteocytes (the number of cells was 31, and the area of a cell was 37 μm${}^{2}$), and in each µ-room, there was at least one osteocyte, which indicates its high secondary stability.

## Figures and Tables

**Table 1:** EDX-analysis of untextured (control) and laser-textured implants.

Sample	Ti	O	Al	V	Total
Control	89.41	0.21	5.56	4.82	100
S	73.16	16.67	7.10	3.07	100
G	69.81	21.08	6.46	2.65	100
I	63.17	27.41	7.00	2.42	100

## Data Availability

Not applicable.

## Abbreviations

The following abbreviations are used in this manuscript:

BIC	bone-to-implant contact
FIC	fibrous-to-implant contact
SEM	scanning electron microscopy
EDX	energy dispersive X-ray
